# Preoperative bimodal imaging evaluation in finding histological correlations of *in situ*, superficial spreading and nodular melanoma

**DOI:** 10.3389/fmed.2024.1436078

**Published:** 2024-08-09

**Authors:** Mircea Negrutiu, Sorina Danescu, Theodor Popa, Liliana Rogojan, Stefan Cristian Vesa, Adrian Baican

**Affiliations:** ^1^Department of Dermatology, “Iuliu Hatieganu” University of Medicine and Pharmacy, Cluj-Napoca, Romania; ^2^Department of Rehabilitation, “Iuliu Hatieganu” University of Medicine and Pharmacy, Cluj-Napoca, Romania; ^3^Department of Histopathology, Cluj-Napoca Emergency County Hospital, Cluj-Napoca, Romania; ^4^Department of Functional Sciences, Discipline of Pharmacology, Toxicology and Clinical Pharmacology, Faculty of Medicine, “Iuliu Hatieganu” University of Medicine and Pharmacy, Cluj-Napoca, Romania

**Keywords:** dermoscopy, melanoma, diagnosis, imaging melanoma, imaging, ultrasound

## Abstract

**Background:**

The aim of this study is to correlate the diagnostic criteria described in dermoscopy, ultrasonography (US), and histology of the most common types of cutaneous melanoma (CM).

**Methods:**

We conducted a prospective study including 40 CM cases, which were analyzed by dermoscopy using the Delta 30 dermatoscope and Vidix 4.0 videodermoscope, by ultrasound (US) using a high-resolution 20 MHz linear probe, along with histopathological analysis.

**Results:**

The study involved 40 patients with histopathologically confirmed CM, comprising 10 nodular melanomas (NM), 21 superficial spreading melanomas (SSM), and nine *in situ* melanomas (MIS). US measurements of tumor thickness exhibited strong correlations with the histopathological Breslow index (BI), particularly in the NM and SSM groups. A notable correlation was observed between the presence of ulceration in histopathology and ultrasonography. Dermoscopic analysis revealed significant associations between specific features and CM types. For instance, the presence of an atypical network, irregular globules, irregular dots, prominent skin margins, angulated lines/polygons, dotted and short linear vessels, and negative network correlated with a median BI ≤ 0.5 mm. Conversely, the presence of blue–white veil, atypical vessels, blue–black color, and milky red color were associated with a median BI ≥ 2.3 mm. Furthermore, regression observed in histopathology correlated with regression identified in dermoscopy, we also found statistical correlations between the presence of vascularization at US with the high Clark level, and the presence of prominent skin markings at dermoscopy. The presence of histopathological regression was more frequently associated with tumors that had precise margins, absent vascularization and with those that did not have ulceration on US. The high mitotic rate was associated with tumors that presented imprecise margins, increased vascularization and US detectable ulceration.

**Conclusion:**

Innovative CM diagnosis using non-invasive methods like dermoscopy and ultrasound may enhance accuracy and treatment guidance by assessing lesion characteristics.

## 1 Introduction

Cutaneous melanoma (CM), is one of the most aggressive forms of skin cancer, marked by its potential to metastasize with devastating consequences and is a significant global health concern (1).

The incidence of CM has been steadily increasing worldwide, especially in regions with high levels of ultraviolet (UV) radiation exposure. While fair-skinned individuals are at a higher risk, melanoma can affect individuals across all racial and ethnic groups. While 50–60 new cases per 100,000 are reported in Australia, the incidence rate in Europe is 10–25 new cases per 100,000, with a notable increase in recent years (2).

Prolonged exposure to UV radiation, remains the most likely cause for melanoma development. Additionally, genetic predispositions, environmental factors, immune suppression, and a history of severe sunburns contribute to the multifaceted etiology of this malignancy (3).

CM can manifest in various clinical forms, with distinct characteristics that may influence prognosis and treatment decisions. When restricted to the epidermis, CM is categorized as melanoma *in situ* (MIS); when atypical melanocytes gradually infiltrate the dermis, it is categorized as invasive melanoma. Four major clinic-pathological subtypes of invasive melanoma have been identified: superficial spreading melanoma (SSM) (41%), nodular melanoma (NM) (16%), lentigo malignant melanoma (LMM) (2.7–14%) and acral lentiginous melanoma (ALM) [1–5% in non-hispanic white population and higher rates in Asian or African American population, to which are added desmoplastic melanoma (1–4%) and amelanotic or hypomelanotic melanoma] (4, 5). Clinical forms of melanoma highlight the diverse presentations of this malignancy, emphasizing the importance of early detection, accurate diagnosis, and appropriate management for each subtype.

The diagnostic process for melanoma involves a combination of clinical evaluation, dermoscopy, and histopathological examination. Histopathological examination is the gold standard for diagnosing melanoma, determining its aggressiveness, and guiding treatment decisions (6). The Breslow index (BI) measures vertical thickness, and correlates with the probability of metastasis (7). Other histological aspects that must be taken into consideration are the presence of ulceration, mitosis rate and the Clark level of invasion. For a better prognosis, a rapid diagnosis is essential in order to initiate the therapeutic attitude (7, 8). Dermoscopy is an invaluable tool in the early detection, diagnosis, and management of melanoma. Its use alongside clinical examination and histopathological evaluation enhances diagnostic accuracy and improves patient outcomes by enabling timely intervention and appropriate treatment (9). Dermoscopy has been used in dermatological evaluation for many years and has proven an increase in diagnostic sensitivity to 90% compared to the naked eye (74%) in the case of melanoma (10). Starting from the classic criteria proposed in 1985 (the ABCD rule), considerable discoveries and progress have been made in establishing the dermatoscopic diagnostic criteria for cutaneous melanoma (11). Recent data shows that the distinct subtypes of melanoma present specific characteristics that can predict the histological diagnosis with different degrees of specificity and sensitivity. Relatively recent, particular criteria have been described for melanoma *in situ* (12), and for differentiating between melanoma and atypical nevus (13). The current research aims to identify the most distinctive aspects of different CM subtypes, capable of predicting various histological aspects as well as the degree of tumor aggressiveness with the highest possible accuracy (14).

Cutaneous ultrasonography (US) complements the clinical examination and is successfully used in the evaluation of CM, providing important information about tumor margins, progression, regression, axes, local invasion, stiffness and micro-vascularization (15).

US represents the first therapeutic approach used in the monitoring of lymph nodes, to detect recurrences or metastases, with a sensitivity superior to the physical examination (16). It is also a useful technique in assisting biopsies, as well as in fine-needle aspiration cytology (US-FNAC), with the aim of anticipating the need to perform the sentinel lymph node excision (17). The use of high-frequency US probes allows for the identification with increased accuracy of the different layers of the skin, permitting accurate determinations of tumor thickness with increasingly better correlations with the histological Breslow index (18).

The combination of dermoscopy and ultrasound addresses many clinical and diagnostic challenges associated with CM by enhancing diagnostic accuracy, providing non-invasive and comprehensive evaluation, guiding biopsy decisions, and facilitating ongoing monitoring and treatment planning. This integrated approach represents a significant advancement in the non-invasive diagnosis and management of cutaneous melanoma, potentially improving patient outcomes and optimizing care.

Other non-invasive imaging techniques include reflectance confocal microscopy (RCM) and line-field confocal optical coherence tomography (LC-OCT), which show high potential in increasing the sensitivity and specificity of clinical diagnosis (19, 20). The treatment for melanoma is often individualized based on the specific characteristics of the tumor and the patient’s overall health. A multidisciplinary approach involving dermatologists, surgical oncologists, medical oncologists, radiation oncologists, and other specialists employed to develop a comprehensive treatment plan (21).

The aim of this study is to correlate the diagnostic criteria described in dermoscopy, US and histopathology of *in situ*, superficial spreading and nodular melanoma.

## 2 Materials and methods

We conducted a prospective study on melanocytic lesions with high suspicion of CM from July 2022 to March 2024, within the Dermatology department of our institution. The selection of the initial 50 patients was based on all available patients that showed melanocytic lesions with high suspicion of CM and were willing to participate in the study. Of the 50 patients selected for the study, 45 remained after the application of the inclusion criteria (suspicious lesion for NM, SSM or MIS, tumors that were to be excised) and 40, respectively, after the application of the exclusion criteria (histological negative result of CM, patients who did not perform tumor excision, patients in whom imaging examinations could not be performed for various reasons). The study was approved by the ethics committee of the University and the local hospital with ID number of protocol: DEP 154/9 May 2022. All study participants were provided with a comprehensive overview of the study protocol and eligibility requirements, and they provided written consent to participate in this study prior to their inclusion. The patients included in the study were aged between 30 and 91 years with an average age of 64 years. The subjects were initially evaluated preoperatively clinically and by non-invasive imaging (US and dermoscopy). The clinical aspects followed were: age, sex, tumor location, clinical diameter, phototype and mode of appearance (*de novo* or nevus-associated).

### 2.1 Ultrasound acquisition

The ultrasonographic data were obtained after the clinical examination by two experienced dermatologists using high-frequency ultrasound (HFUS) (Philips Affiniti). A high-resolution 20 MHz linear probe was used as it provides satisfactory accuracy for skin analysis. Although it falls short in detailing the epidermal ultrastructure, which is confirmed by its limited efficacy in evaluating melanoma *in situ* (MIS), it enables effective assessment of the dermis, hypodermis, and even the muscular fascia. Therefore, this frequency offers favorable penetration for evaluating cutaneous tumor pathology. Tumors were assessed using both thyroid and cutaneous modes. Both B mode and color Doppler were performed for all lesions. The evaluation of the tumors was carried out with the probe perpendicular to them, applying as little pressure as possible and with a sufficient amount of gel, so that the tumor is completely covered. We evaluated the shape (regular/irregular), the tumor thickness, and the level of invasion, the margins (precise/imprecise), the presence of vascularization, the number of vascular pedicles and the presence of ulceration. The thickness was measured on two axes (longitudinal and transverse) starting at the granular layer (below the stratum corneum’s hyperechoic band) and proceeding to the deepest point of visible tumor infiltration.

We divided the level of tumor invasion into five levels of invasion according to the Clark level.

Using the color Doppler mode, the kind of tumor vascularization and the quantity of vascular pedicles were investigated. We graded tumor vascularity according to the number of vascular pedicles, from 0 to 3. We considered 0-no pedicle, 1-a main central pedicle, 2-a main central pedicle and at least one secondary lateral pedicle, 3-a main central pedicle and two secondary lateral pedicles.

### 2.2 Dermoscopic evaluation

Using both the Delta 30 dermatoscope and the Vidix 4.0 videodermatoscope with Vectra software (Version 7.4.7), in conventional and polarized light, the dermoscopic pictures were assessed by the same two experienced dermatologists who were blind to the diagnosis.

The selection of dermoscopic criteria was inspired by the work of Lallas et al., because it includes a specific analysis of them from the histological point of view, the localization and the histological subtype (14).

For the entire group of patients, we followed: global pattern (reticular, globular, structureless, starburst, and multicomponent), criteria corresponding to junctional alterations (atypical network, irregular globules, irregular dots, pseudopods, irregular blotch, irregular hyperpigmented areas, prominent skin margins, angulated lines/polygons, dotted and short linear vessels), criteria corresponding to mixed junctional/dermal alterations (regression structures, negative network, white shiny streaks), criteria corresponding to dermal alterations (blue–white veil, atypical vessels), blue–black color, milky red coloration.

### 2.3 Histological evaluation

Following the clinical and paraclinical imaging examination, the melanocytic tumors were excised according to the hospital protocol with 0.2 cm margins and examined histopathologically. The tumor tissue was fixed with hematoxylin–eosin and examined under an optical microscope. The following criteria were followed: histological type, Breslow index, Clark level of invasion, presence of ulceration, perineural invasion, angiolymphatic invasion, mitotic rate, presence of satellite lesions, and presence of regression.

### 2.4 Statistical analysis

Statistical analysis was carried out using the MedCalc® Statistical Software version 22.021 (MedCalc Software Ltd., Ostend, Belgium; 2024).^1^ Data were expressed as median and interquartile range or frequency and percentage, as they had a non-normal distribution (tested by Shapiro–Wilk test). Comparisons between measurements were carried out using the Mann–Whitney test or chi-square test, as appropriate. The Mann–Whitney test was chosen for comparing non-normally distributed continuous variables because it does not assume normality, making it suitable for skewed distributions. The chi-square test was used for comparing categorical variables, as it assesses the association between two categorical variables and is robust to the distribution shape of the data, provided the sample size is sufficiently large. To determine which variables are independently associated with a binary variable, we introduced variables that achieved statistical significance in univariate analysis into a logistic regression model and chose the most stable model. For identifying variables independently associated with a quantitative variable, we introduced variables that achieved statistical significance in univariate analysis into a linear regression model and selected the most stable model. A *p*-value <0.05 was considered statistically significant.

## 3 Results

The study included a number of 40 patients with histopathologically confirmed CM. Nodular melanomas (10), superficial spreading melanoma (21), and *in situ* melanoma (9) were included, of which 15 (37.5%) were women and 25 (62.5%) were men. The main phototype was type 3 found in 29 (72.5%) of the patients. Regarding the mode of appearance, 21 (52.5%) were *de novo* and 19 (47.5%) were nevus-associated. The patients’ age ranged from 30 to 91 years, with an average of 64. The preferred location was the chest (13), followed by the head region (6) and the leg (5).

### 3.1 Histopathology

Histologically, 2 (5%) presented a satellite lesion, 11 (27.5%) regression, 2 (5%) perineural invasion, 8 (20%) ulceration, 1 (2.5%) angiolymphatic invasion. The BI ranged from 0 mm (for MIS) to 24 mm with a mean of 2.5 mm. The histopathological characteristics depending on the subtype of CM are highlighted in Table 1.

**Table 1:** The histopathological characteristics depending on the subtype of CM.

		SSM	MIS	NM	Total
Satellite lesion		0	0	2	2 (5%)
Regression		7	2	2	11 (27.5%)
Perineural invasion		1	0	1	2 (5%)
Ulceration		3	0	5	8 (20%)
Angiolymphatic invasion		0	0	1	1 (2.5%)
Clark	I	0 (0%)	9 (100%)	0 (0%)	9 (22.5%)
	II	6 (28.6%)	0 (0%)	0 (0%)	6 (15%)
	III	9 (42.9%)	0 (0%)	0 (0%)	9 (22.5%)
	IV	6 (28.6%)	0 (0%)	8 (80%)	14 (35%)
	V	0 (0%)	0 (0%)	2 (20%)	2 (5%)
Breslow index		0.7	0.0	5.1	–
Mitotic rate		1	0	8	–

### 3.2 Dermoscopy

We analyzed the presence of dermatoscopic criteria for each histological subtype of melanoma (Table 2). The most frequent criteria for SSM were the presence of an atypical network (90%), irregular hyperpigmented areas (95.2%), irregular blotch (61.9%) and white shiny streaks (61.9%). For MIS, the representative criteria were the presence of an atypical network (100%), irregular hyperpigmented areas and angulated lines/polygons (66.7%). NM was characterized by irregular hyperpigmented areas (90%), blue–white veil (100%), atypical vessels (100%), and milky red coloration (90%).

**Table 2:** Dermatoscopic criteria for each histological subtype of melanoma.

Variable		SSM	MIS	NM	Total	*p*
Global DSC pattern	Globular	4 (19%)	3 (33.3%)	0 (0%)	7 (17.5%)	<0.001
	Multicomponent	11 (52.4%)	1 (11.1%)	0 (0%)	12 (30%)	
	Reticular	3 (14.3%)	3 (33.3%)	0 (0%)	15 (15%)	
	Starburst	3 (14.3%)	2 (22.2%)	0 (0%)	5 (12.5%)	
Atypical network		19 (90%)	9 (100%)	1 (10%)	29 (72.5%)	<0.001
Irregular globules		12 (57.1%)	4 (44.4%)	0 (0%)	16 (40%)	0.010
Irregular dots		5 (23.8%)	5 (55.6%)	1 (10%)	11 (27.5%)	0.073
Pseudopods		7 (33.3%)	1 (11.1%)	0 (0%)	8 (20%)	0.071
Irregular blotch		13 (61.9%)	5 (55.6%)	1 (10%)	19 (47.5%)	0.022
Irregular hyperpigmented areas		20 (95.2%)	8 (88.9%)	9 (90%)	37 (92.5%)	0.784
Prominent skin markings		4 (19%)	4 (44.4%)	1 (10%)	9 (22.5%)	0.172
Angulated lines/polygons		6 (28.6%)	6 (66.7%)	0 (0%)	12 (30%)	0.007
Dotted and short linear vessels		10 (47.6%)	5 (55.6%)	0 (0%)	15 (37.5%)	0.017
Regression structures		9 (42.9%)	4 (44.4%)	3 (30%)	16 (40%)	0.755
Negative network		9 (42.9%)	3 (33.3%)	1 (10%)	13 (32.5%)	0.188
White shiny streaks		13 (61.9%)	3 (33.3%)	3 (30%)	19 (47.5%)	0.157
Blue–white veil		11 (52.4%)	1 (11.1%)	10 (100%)	22 (55%)	<0.001
Atypical vessels		4 (19%)	0 (0%)	10 (100%)	14 (35%)	<0.001
Blue–black color		3 (14.3%)	0 (0%)	10 (100%)	13 (32.5%)	<0.001
Milky red		1 (4.8%)	0 (0%)	9 (90%)	10 (25%)	<0.001

We evaluated the presence of dermoscopic criteria according to the tumor thickness (Breslow index) (Table 3). The presence of an atypical network, irregular globules, irregular dots, prominent skin margins, angulated lines/polygons, dotted and short linear vessels, negative network was correlated with median BI ≤ 0.5 mm. The presence of a blue–white veil, atypical vessels, blue–black color and milky red color were associated with median BI ≥ 2.3 mm.

**Table 3:** Dermatoscopic criteria according to the Breslow index.

Variable		Breslow index			*p*
		Percentile 25	Median	Percentile 75	
Atypical network	No	4.20	4.50	10.00	<0.001
	Yes	0.00	0.50	0.90	
Irregular globules	No	0.27	1.60	4.50	0.045
	Yes	0.12	0.50	0.90	
Irregular dots	No	0.50	0.90	4.40	0.008
	Yes	0.00	0.50	0.50	
Pseudopods	No	0.12	0.90	4.27	0.413
	Yes	0.27	0.60	0.98	
Irregular blotch	No	0.35	2.50	5.15	0.001
	Yes	0.00	0.70	0.90	
Irregular hyperpigmented areas	No	0.00	0.20	–	0.534
	Yes	0.50	0.90	3.20	
Prominent skin markings	No	0.50	0.90	4.20	0.027
	Yes	0.00	0.50	0.60	
Angulated lines/polygons	No	0.50	1.12	4.45	0.001
	Yes	0.00	0.25	0.65	
Dotted and short linear vessels	No	0.50	1.90	4.50	0.004
	Yes	0.00	0.50	0.70	
Regression structures	No	0.27	0.80	4.12	0.666
	Yes	0.12	0.70	2.20	
Negative network	No	0.20	0.90	4.50	0.130
	Yes	0.25	0.50	0.97	
White shiny streaks	No	0.00	0.90	4.50	0.444
	Yes	0.50	0.70	1.00	
Blue–white veil	No	0.00	0.50	0.55	<0.001
	Yes	0.85	2.30	4.82	
Atypical vessels	No	0.00	0.50	0.75	<0.001
	Yes	2.40	4.40	8.65	
Blue–black color	No	0.00	0.50	0.90	<0.001
	Yes	3.20	4.50	9.10	
Milky red	No	0.00	0.50	0.90	<0.001
	Yes	3.85	5.15	10.5	

The regression observed in histopathology correlated with the regression identified in dermoscopy with a Cohen kappa coefficient of 0.505 (*p* = 0.001).

### 3.3 Ultrasound

The ultrasound appearance of melanomas was described as hypoechoic tumors with a regular shape (57.5%), well-defined margins (60%), without ulceration (77.5%), some being also vascularized (45%). US findings according to the histopathological subtype are presented in Table 4.

**Table 4:** US findings according to the histopathological subtype.

Variable		SSM	MIS	NM	Total	*p*
Shape	Regular	13 (61.9%)	8 (88.9%)	2 (20%)	23 (57.5%)	0.008
	Irregular	8 (38.1%)	1 (11.1%)	8 (80%)	17 (42.5%)	
Margins	Well-defined	13 (61.9%)	8 (88.9%)	3 (30%)	24 (60%)	0.032
	Indistinct	8 (38.1%)	1 (11.1%)	7 (70%)	16 (40%)	
Vascularization	Absent	13 (61.9%)	9 (100%)	0 (0%)	22 (55%)	<0.001
	Present	8 (38.1%)	0 (0%)	10 (100%)	18 (45%)	
No. vascular pedicles	0	13 (61.9%)	9 (100%)	0 (0%)	22 (55%)	<0.001
	1	6 (28.6%)	0 (0%)	1 (10%)	7 (17.5%)	
	2	2 (9.5%)	0 (0%)	1 (10%)	3 (7.5%)	
	3	0 (0%)	0 (0%)	8 (80%)	8 (20%)	
Ulceration	Absent	17 (81%)	9 (100%)	5 (50%)	31 (77.5%)	0.029
	Present	4 (19%)	0 (0%)	5 (50%)	9 (22.5%)	

We found a significant Spearman correlation coefficient for the entire group (*r* = 0.984, *p* < 0.001), indicating a strong association between the measured BI by histology and the calculated US tumor thickness. This suggests that, as validated by histological investigation, the measures made by US nearly matched the actual tumor thickness. The best correlation was achieved for the NM group (*r* = 0.991, *p* < 0.001), and for the SSM (*r* = 0.927, *p* < 0.001). Five (55.5%) out of nine patients with MIS had tumor thickness that could be measured by US.

We observed a good correlation between the presence of ulceration from histopathology and that detected by US, with a Cohen kappa coefficient of 0.925 (*p* < 0.001).

The presence of vascularization varied depending on the histological subtype: absent in the MIS group, present 100% for NM and 38.1% for SSM, with an abundance of vascular pedicles for NM.

We identified a statistically significant presence of imprecise edges, vascularization, high number of pedicles (≤2), ulceration, in tumors with higher BI (illustrated in Table 5).

**Table 5:** The variation of ultrasound criteria depending on the Breslow index.

Variable		Breslow index			*p*
		Percentile 25	Median	Percentile 75	
Margins	Indistinct		2.60		0.017
	Well-defined	0.00	0.60	0.98	
Vascularization	Absent	0.00	0.50	0.75	0.000
	Present	0.97	4.05	6.40	
No. vascular pedicles	0	0.00	0.50	0.75	0.000
	1	0.50	0.90	2.10	
	2	1.90	4.20	–	
	3	4.35	7.00	11.50	
Ulceration	Absent	0.00	0.50	0.95	<0.001
	Present	0.90	2.20	10.10	

Furthermore, we investigated the relationship between the histological Clark level of tumor invasion and the US-determined depth of tumor invasion (epidermis, papillary dermis, reticular dermis, and hypodermis). The variation of US criteria depending on the level of Clark invasion is illustrated in Table 6.

**Table 6:** The variation of ultrasound criteria depending on the level of Clark invasion.

Variable		Clark I	Clark II	Clark III	Clark IV	Clark V	Total	*p*
Margins	Indistinct	1 (11.1%)	2 (33.3%)	3 (33.3%)	8 (57.1%)	2 (100%)	16 (40%)	0.087
	Well-defined	8 (88.9%)	4 (66.7%)	6 (66.7%)	6 (42.9%)	0 (0%)	24 (60%)	
Vascularization		0 (0%)	2 (33.3%)	3 (33.3%)	11 (78.6%)	2 (100%)	18 (45%)	0.002
No. vascular pedicles	0	9 (100%)	4 (66.7%)	6 (66.7%)	3 (21.4%)	0 (0%)	22 (55%)	0.006
	1	0 (0%)	2 (33.3%)	2 (22.2%)	3 (21.4%)	0 (0%)	7 (17.5%)	
	2	0 (0%)	0 (0%)	1 (11.1%)	2 (14.3%)	0 (0%)	3 (7.5%)	
	3	0 (0%)	0 (0%)	0 (0%)	6 (42.9%)	2 (100%)	8 (20%)	
Ulceration		0 (0%)	0 (0%)	2 (22.2%)	5 (35.7%)	2 (100%)	9 (22.5%)	0.013

### 3.4 Statistical correlation

To establish statistical correlations between dermatoscopic, ultrasound, and histological findings, we categorized the patient group into two sections. The first category included non-nodular melanomas (MIS + SSM) and the second category included nodular melanomas (NM). The distribution of ultrasound and dermatoscopic criteria according to this division are illustrated in Table 7.

**Table 7:** The variation of ultrasound and dermoscopy criteria depending on the histological nodular or non-nodular melanoma.

Variable		Non-nodular melanoma (MIS + SSM)	Nodular melanoma (NM)	*p*
**US**				
Margins	Indistinct	9 (30%)	7 (70%)	0.059
	Well-defined	21 (70%)	3 (30%)	
Vascularization	Absent	22 (73.3%)	0 (0%)	0.000
	Present	8 (26.7%)	10 (100%)	
Ulceration	Absent	26 (86.7%)	5 (50%)	0.029
	Present	4 (13.3%)	5 (50%)	
**Dermoscopy**				
Atypical network		28 (93.3%)	1 (10%)	0.000
Irregular globules		16 (53.3%)	0 (0%)	0.003
Irregular dots		10 (33.3%)	1 (10%)	0.233
Pseudopods		8 (26.7%)	0 (0%)	0.165
Irregular blotch		18 (60%)	1 (10%)	0.009
Irregular hyperpigmented areas		28 (93.3%)	9 (90%)	1.00
Prominent skin margins		8 (26.7%)	1 (10%)	0.404
Angulated lines/polygons		12 (40%)	0 (0%)	0.019
Dotted and short linear vessels		15 (50%)	0 (0%)	0.006
Regression structures		13 (43.3%)	3 (30%)	0.711
Negative network		12 (40%)	1 (10%)	0.124
White shiny streaks		16 (53.3%)	3 (30%)	0.281
Blue–white veil		12 (40%)	10 (100%)	0.001
Atypical vessels		4 (13.3%)	10 (100%)	0.000
Blue–black color		3 (10%)	10 (100%)	0.000
Milky red		1 (3.3%)	9 (90%)	0.000

In order to find out which variables are independently associated with Clark level, we introduced several variables in a logistic model. The presence of vascularization at ultrasound was associated with a high Clark level (OR – 19.5, CI 95% 1.7–216.2; *p* = 0.01), and the presence of prominent skin markings at dermoscopy was less likely to be associated with a high Clark level (OR – 0.085; CI 95% 0.008–0.957; *p* = 0.04). Irregular dots were not significantly associated with the Clark level. The distribution of dermatoscopic and ultrasound criteria according to the Clark level are presented in Table 8.

**Table 8:** The distribution of dermatoscopic and ultrasound criteria according to the Clark level.

Variable		Clark level I–II	Clark level III–IV–V	*p*
**US**				
Margins	Indistinct	3 (20%)	13 (52%)	0.096
	Well-defined	12 (80%)	12 (48%)	
Vascularization	Absent	13 (86.7%)	9 (36%)	0.005
	Present	2 (13.3%)	16 (64%)	
Ulceration	Absent	15 (100%)	16 (64%)	0.025
	Present	0 (0%)	9 (36%)	
**Dermoscopy**				
Atypical network		15 (100%)	14 (56%)	0.003
Irregular globules		7 (46.7%)	9 (36%)	0.739
Irregular dots		8 (53.3%)	3 (12%)	0.009
Pseudopods		4 (26.7%)	4 (16%)	0.683
Irregular blotch		9 (60%)	10 (40%)	0.369
Irregular hyperpigmented areas		13 (86.7%)	24 (96%)	0.545
Prominent skin margins		7 (46.7%)	2 (8%)	0.008
Angulated lines/polygons		8 (53.3%)	4 (16%)	0.030
Dotted and short linear vessels		7 (46.7%)	8 (32%)	0.555
Regression structures		8 (53.3%)	8 (32%)	0.317
Negative network		7 (46.7%)	6 (24%)	0.257
White shiny streaks		7 (46.7%)	12 (48%)	1.00
Blue–white veil		4 (26.7%)	18 (72%)	0.014
Atypical vessels		0 (0%)	14 (56%)	0.001
Blue–black color		0 (0%)	13 (53%)	0.002
Milky red		0 (0%)	10 (40%)	0.014

The high mitotic rate was associated with tumors that presented imprecise margins (*p* = 0.016), increased vascularization (*p* = 0.000) and US detectable ulceration.

The presence of histopathological regression was more frequently associated with tumors that had precise margins (*p* = 0.728), absent vascularization (*p* = 1.00) and with those that did not have ulceration (0.227) on US.

In order to find out which variables are independently associated with mitotic rate, we introduced several variables in a linear model. The presence of atypical network at dermoscopy was less likely to be associated with high mitotic rate (*t* = −4.64 (CI 95% −15.64; −6.13); *p* < 0.001). The presence of ulceration at ultrasound and milky red color at dermoscopy were not significantly associated with mitotic rate (*p* = 0.1; *p* = 0.1, respectively). The distribution of mitotic rate and regression according to dermatoscopic criteria is presented in Table 9.

**Table 9:** The distribution of mitotic rate and regression according to dermatoscopic criteria.

Variable	Mitotic rate (percentile)			*p*	Regression	*p*
	25	50	75			
Atypical network	0.00	1	2	0.000	9 (81.8%)	0.694
Irregular globules	0.00	1	1.75	0.013	3 (27.3%)	0.473
Irregular dots	0.00	0.00	1	0.012	1 (9.1%)	0.233
Pseudopods	0.00	1	1.75	0.105	2 (18.2%)	1.00
Irregular blotch	0.00	1	2	0.006	7 (63.6%)	0.366
Irregular hyperpigmented areas	0.00	1	5	0.753	10 (90.9%)	1.00
Prominent skin margins	0.00	1	1	0.129	2 (18.2%)	1.00
Angulated lines/polygons	0.00	1	1	0.011	3 (27.3%)	1.00
Dotted and short linear vessels	0.00	1	1	0.024	7 (63.6%)	0.65
Regression structures	0.25	1	4.25	0.527	9 (81.8%)	0.003
Negative network	0.00	1	1.5	0.022	3 (27.3%)	1.00
White shiny streaks	1	1	3	0.392	5 (45.5%)	1.00
Blue–white veil	1	4.5	8.25	0.001	7 (63.6%)	0.723
Atypical vessels	4.75	7	19.25	0.000	4 (36.4%)	1.00
Blue–black color	4.50	6	14.50	0.000	4 (36.4%)	1.00
Milky red	5	7	20.5	0.000	3 (27.3%)	1.00

## 4 Discussion

US is a useful tool in the overall care of melanoma since it helps with disease monitoring, diagnosis, staging, and therapy planning (22, 23). It enhances clinical evaluations and other imaging modalities, improving patient outcomes and enabling more informed healthcare decisions (24).

In a series of studies in literature, correlations were sought between the thickness of the melanoma and the BI in histology. To increase the accuracy of the technique, probes from 12 MHz to 100 MHz were used. We obtained a correlation of 0.984 for the entire group of melanomas, using a 20 MHz probe. The best correlation was obtained for the group of NM (*r* = 0.991) and SSM (*r* = 0.927). Similar correlations were obtained in other studies in literature (25). The mean BI for the NM group was 5.1 mm, and for SSM 0.7 mm, suggesting a better correlation for thicker tumors. The work by Kaikaris et al. (26) showed that the 14 MHz probe obtains better correlations for tumors with a thickness greater than 2 mm. Despite the apparently good correlation, there were submillimeter differences between histological and ultrasound measurements, especially in cases of thin melanomas. For MIS, in 5 (55.5%) of nine cases, ultrasound-measurable tumor thickness was identified. A series of studies showed this tendency to overestimate the thickness of melanomas using the 20 MHz probe, but still with a strong correlation with histopathology (27). A drawback of 20 MHz US is that tumor thickness might be overestimated due to lymphocytic infiltrates, regression, and naevus remnants (15).

A study by Hinz et al. (28) shows the superiority of OCT in determining the tumor thickness compared to HFUS (20 MHz), but on a limited group of patients confirmed with CM. Even with its improved resolution, OCT has a limited penetration depth and is unable to measure the thickness of lesions larger than 1 mm, such as nevi or aggressive melanomas (29).

One clinically and prognostically relevant characteristic of melanoma that is linked to aggressive tumor behavior and unfavorable outcomes is ulceration. Accurate risk assessment and treatment planning for melanoma patients depend on its identification and integration into staging systems (30). Histological ulceration was present in 8 (20%) of the cases and correlated well with US (*k* = 0.925). This varied directly proportional to the thickness of the BI, being absent in those with a median of 0.5 mm and present in those with a median of 2.20 mm, suggesting a negative prognosis factor.

Although studies in the literature are still inconsistent, the presence of microvessels in a skin lesion enhances the likelihood of malignancy. Microvessel identification has demonstrated an excellent specificity for malignancy (90–100%), although its sensitivity has been in the range of 34–100% (31). In our study, the presence of vascularization was identified in 18 (45%) of the cases and varied directly proportional to the thickness of the Breslow index, being absent in those with a median thickness of 0.5 mm. The vascular density expressed in the number of pedicles was significantly higher in the NM group. Faita et al. (32) using ultra-high frequency ultrasound (UHFUS) (70 MHz) showed that the intralesional vascularization is not sufficient for the differentiation of NM from melanocytic nevi. Another recent study proposes the detection of sublesional vessels (UHFUS 70 MHz), which are highlighted as tortuous hypoechoic structures, an aspect not encountered by us, more likely due to the impossibility of detection with the 20 MHz probe (33).

Satellite melanoma metastases are described as hypoechoic subcutaneous nodules, with diminished intralesional echo, irregular or polycyclic edges, and may have posterior amplification. Larger lesions may present anechoic areas corresponding to areas of necrosis (34). In our group, there were two tumors with satellite metastases, both identified by ultrasound.

**Figure 1:**
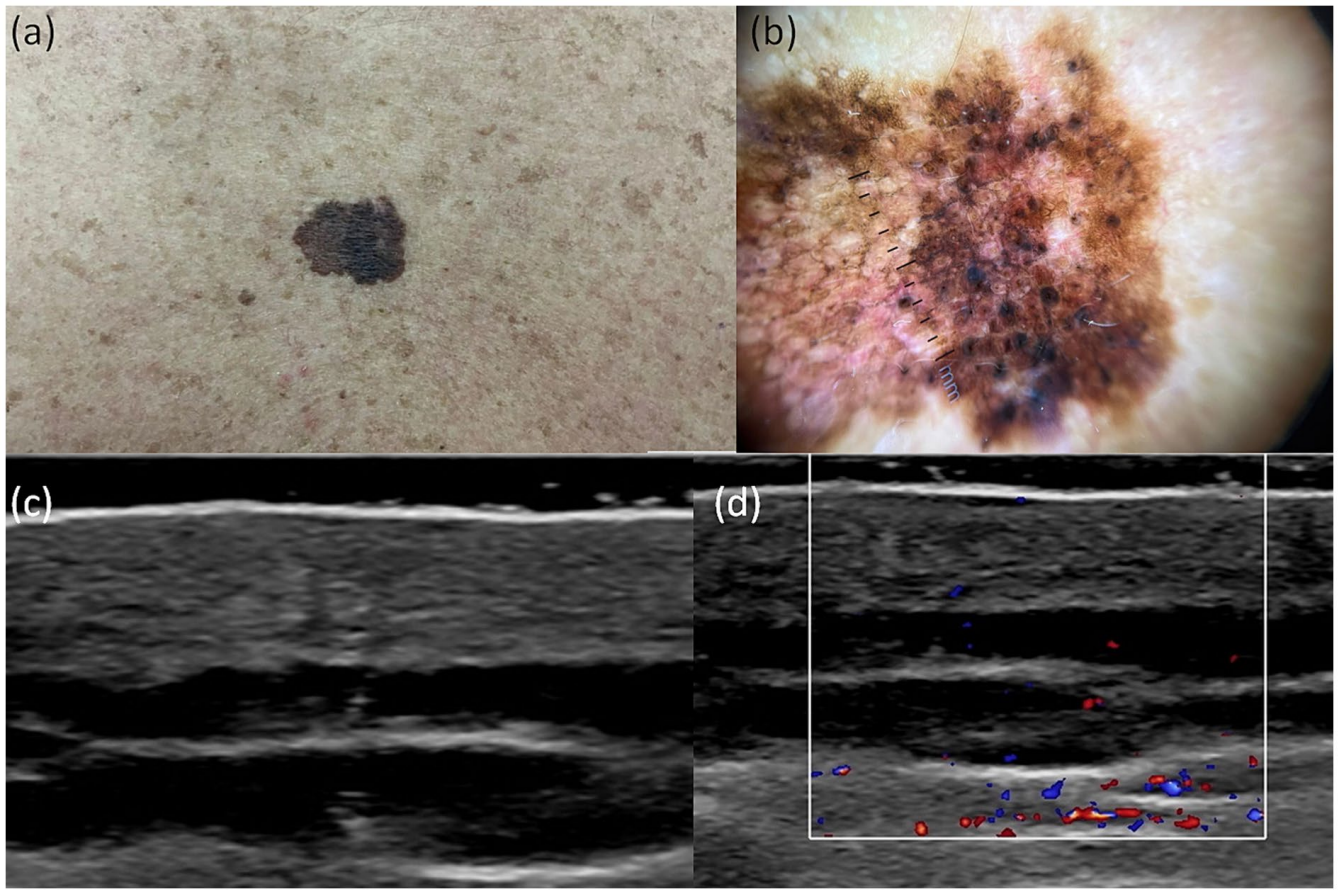
Clinical picture of MIS **(A)**. Dermatoscopy shows the presence of an atypical network with hyperpigmented areas, angulated lines, regression structures, and irregular dots **(B)**. US shows a hypoechoic, oval, ill-defined lesion that reaches the level of the epidermis, loss of the bilaminar pattern of the epidermis **(C)**. Doppler mode shows no sign of regional hypervascularity **(D)**.

**Figure 2:**
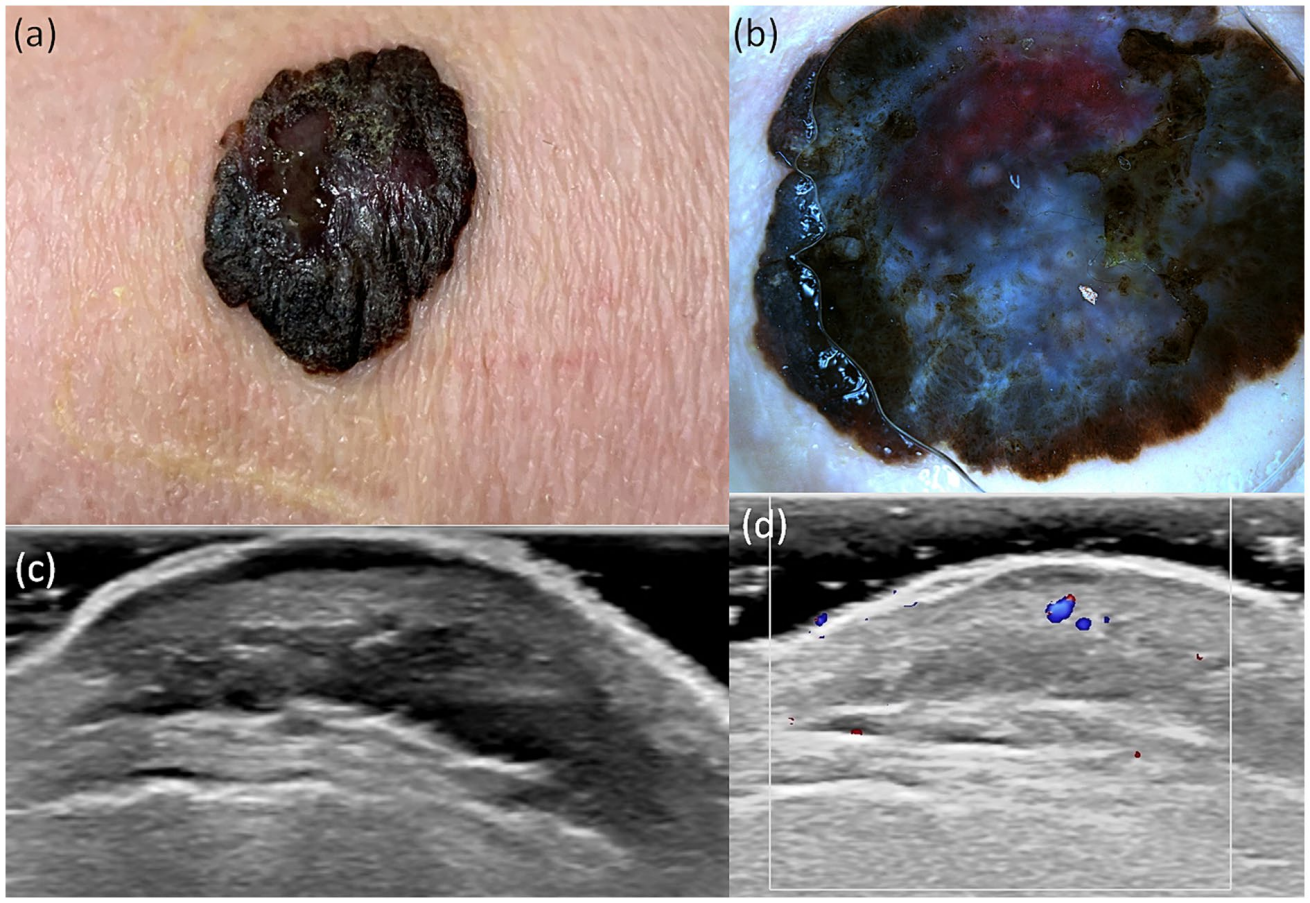
Clinical picture of SSM **(A)**. Dermatoscopy shows the presence of an atypical network, irregular hyperpigmented areas, irregular blotch, pseudopods, atypical vesseles, and blue–black color **(B)**. US shows a hypoechoic, oval, ulcerated, well-defined lesion that reaches the dermis **(C)**. Doppler mode shows an increase in tumor vascularity **(D)**.

**Figure 3:**
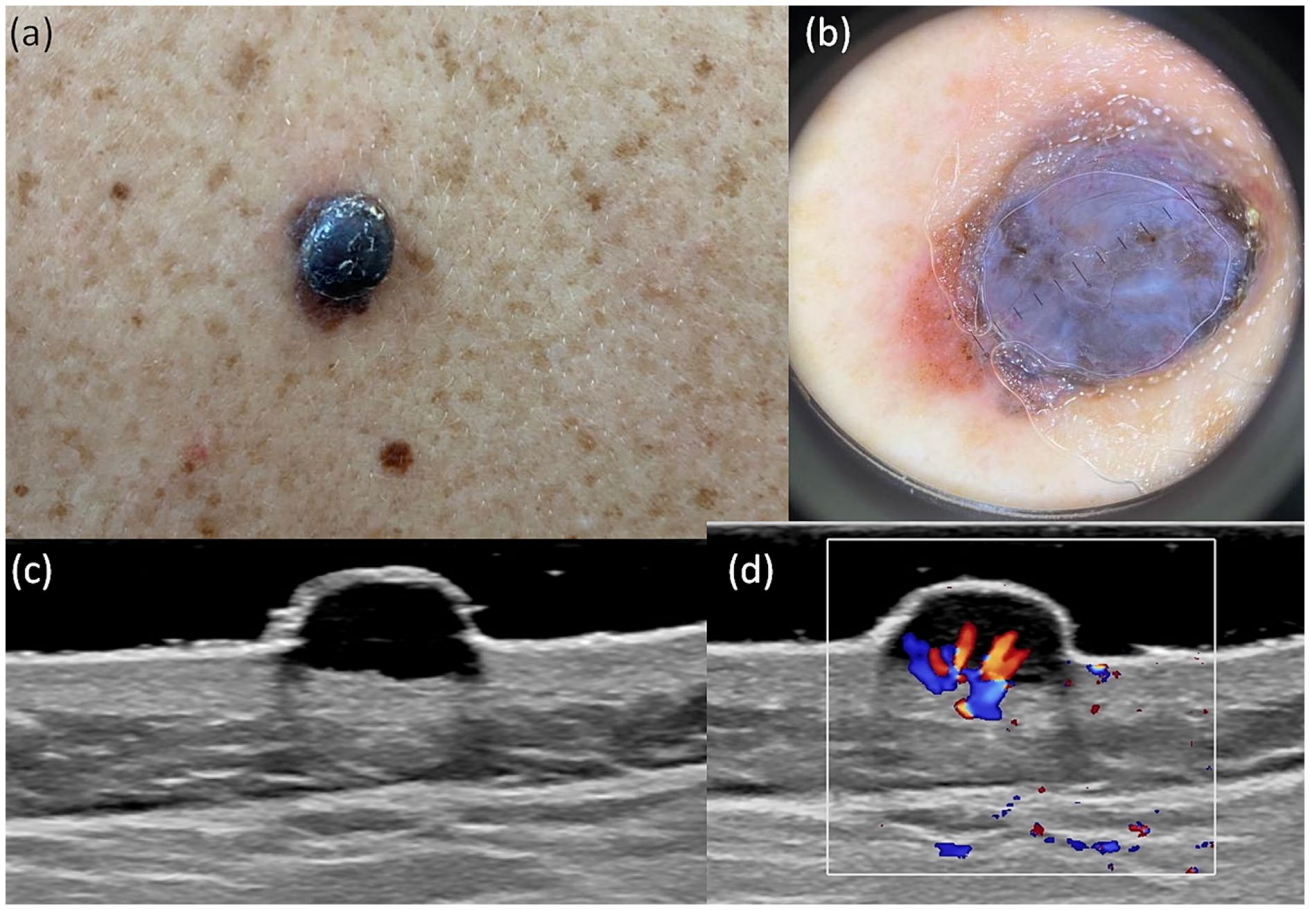
Clinical picture of MN **(A)**. Dermatoscopy shows the presence of a nodular tumor with blue–white veil, with atypical vessels on surface, and blue–black color **(B)**. US shows a hypoechoic, oval, well-defined lesion that reaches the level of the reticular dermis **(C)**. Doppler mode shows an increase in tumor vascularity **(D)**.

A useful technique for the early identification and detection of melanoma is dermoscopy. In our study aiming to establish a bimodal algorithm for the preoperative evaluation of suspicious melanoma lesions, we selected the criteria proposed by Lallas et al. (14). We evaluated three subtypes of melanoma: MIS (Figure 1 shows the clinical, dermoscopic, and US features), SSM (Figure 2), and NM (Figure 3). For SSM, the most frequently encountered criteria were the presence of an atypical network, irregular hyperpigmented areas, white shiny streaks, similar data suggesting Nadeem et al. (35).

For MIS the most frequent criteria were atypical network and irregular hyperpigmented areas and for NM there was the presence of a blue–white veil, atypical vessels and blue–black color.

A study by Lallas et al. (12) showed that the most frequent criteria associated with MIS, compared to excised nevi, are irregular hyperpigmented areas and prominent skin margins. The last criterion was present in 44.4% of cases in our MIS group. In several studies, an association was observed between the presence of an atypical network and negative sentinel lymph node (SLN) and the presence of ulceration and irregular blotch with positive SLN (36, 37). In this context, we found a more specific association of atypical network with tumors whose median BI was 0.5 mm and for the presence of irregular blotch the median BI was 0.7 mm.

Rodríguez-Lomba et al. (38) suggest an association between melanomas with a thickness greater than 0.8 mm and the presence of a blue–white veil, blue–black color, and milky red color, a finding also observed in our research, being the most present among NM with a median IB greater than 4.4 mm. Studies have proved that the presence of dotted vessels within a nodular formation indicates a high probability of a NM (39). We classified this type of vascularization in atypical vessels, which was more frequently associated with NM, with a median BI of 4.4 mm and a Clark invasion level higher than III.

Regression in melanoma represents a complex histopathological phenomenon associated with immune-mediated destruction of tumor cells. While its prognostic significance remains debated, regression is an important histopathological feature to recognize and evaluate in the management of melanoma patients (40). We found a correlation of 0.505 between the regression detected dermoscopically and the histological result, with a presence in 40% of tumors and an association with those whose median BI was 0.8 mm.

In this study we proposed a bimodal imaging approach of the patient with preoperative cutaneous melanoma. Dermoscopy provided detailed images of the tumor surface, being able to predict the tumor subtype, but had limitations in depth assessment. US completed with information such as tumor thickness, vascularization, local invasion, satellite metastases, but also the relationship with neighboring structures. The combined use of the two techniques offered the possibility of holistic, *in vivo* investigation of cutaneous melanomas.

The correlation between dermoscopy, ultrasound, and histopathology provides insight for potential strategies in choosing the best diagnosis approach for minimum invasivity without increasing the risk of misdiagnosis. While the histopathological analysis is the gold standard, it is an irreversible procedure, with potential side effects such as scars. A high correlation between a US finding, or a dermoscopy feature, with a histopathological finding, means that we can be more certain in our decision to remove the lesion or to leave the lesion alone. However, this paper will not go beyond the reporting of correlations, as a diagnosis protocol requires much more data and risk analysis.

This combined approach underscores the novelty of our research, wherein the integration of dermatoscopic, ultrasound, and histological data provides a more comprehensive understanding of melanoma diagnosis. We found statistical correlations between the presence of vascularization at US with the high Clark level, and the presence of prominent skin markings at dermoscopy. The presence of histopathological regression was more frequently associated with tumors that had precise margins, absent vascularization and with those that did not have ulceration on US. The high mitotic rate was associated with tumors that presented imprecise margins, increased vascularization and US detectable ulceration. By elucidating independent associations and correlations across multiple diagnostic modalities, our findings contribute to refining diagnostic protocols and potentially enhancing clinical outcomes in melanoma management.

In conclusion, this study not only validates existing correlations but also introduces a novel perspective through the integration of multiple diagnostic criteria. Future research can build upon these insights to further refine diagnostic algorithms and improve patient care strategies in melanoma detection and management.

### 4.1 Limitations

Our study faced a number of limitations. First of all, dermoscopy and skin ultrasound are subjective investigations, a fact that we tried to prevent through independent evaluation by two experienced investigators. Ultrasound tumor thickness measurement does not have a standard protocol, so it is subjected to error. A series of studies showed the tendency to overestimate the thickness of melanomas using the 20 MHz probe, but still with a strong correlation with histopathology. A drawback of 20 MHz US is that tumor thickness might be overestimated due to lymphocytic infiltrates, regression, and naevus remnants.

Additionally, we included a relatively small number of patients with uneven distribution for each subtype. Only three subtypes of melanoma (SSM, NM, and MIS) were included, so our study does not include data on LMM, ALM, desmoplastic melanoma, and amelanotic melanoma. The subtypes not included are less frequent and would have increased the error rate of the statistical analysis.

### 4.2 Future perspectives

Combining dermoscopy and ultrasound imaging modalities can provide complementary information about melanocytic lesions. The development of integrated imaging systems that enable the simultaneous or sequential capture of US and dermoscopic images may be the main focus of future research. By combining the advantages of both modalities, an integrated method may increase diagnostic accuracy by offering a more thorough evaluation of lesion form, depth, and vascularity.

Clinical decision assistance in real time may be made possible by integrating machine learning algorithms with dermoscopy and US equipment. Based on the examination of imaging data and patient-specific variables, these systems might offer automated lesion assessment, risk prediction, and tailored therapy recommendations. Enhancing patient care, streamlining clinical workflows, and increasing diagnostic accuracy are all possible with real-time decision assistance.

## 5 Conclusion

This study reveals key insights into the correlations between diagnostic criteria observed in dermoscopy, ultrasonography (US), and histology for different types of cutaneous melanoma (CM). As the first study to simultaneously evaluate dermoscopy and ultrasound findings in relation to histology, it offers a more comprehensive assessment of CM. Our findings suggest that integrating these diagnostic methods can guide the selection of less invasive diagnostic strategies without compromising accuracy. Although histopathology remains the definitive diagnostic standard, it involves irreversible procedures and can lead to unaesthetic scars. The high correlation observed between US and dermoscopic features with histopathological findings enhances confidence in clinical decisions regarding whether to excise or monitor lesions.

## Data availability statement

The original contributions presented in the study are included in the article/supplementary material, further inquiries can be directed to the corresponding author.

## Ethics statement

The studies involving humans were approved by the Ethics Committee of University of Medicine and Pharmacy “Iuliu Hatieganu” Cluj-Napoca (ID number of the protocol: DEP153/9 May 2022). The studies were conducted in accordance with the local legislation and institutional requirements. The participants provided their written informed consent to participate in this study.

## Author contributions

MN: Data curation, Formal analysis, Methodology, Project administration, Writing – original draft. SD: Conceptualization, Methodology, Resources, Writing – review & editing. TP: Software, Writing – original draft. LR: Methodology, Writing – review & editing. SV: Data curation, Formal analysis, Validation, Writing – review & editing. AB: Resources, Supervision, Visualization, Writing – review & editing.

## Footnotes

^1^ https://www.medcalc.org
